# Characteristics and mortality of severe influenza cases treated with parenteral aqueous zanamivir, United Kingdom, October 2009 to January 2011

**DOI:** 10.1111/irv.12603

**Published:** 2018-10-19

**Authors:** Paul Robert Cleary, Jonathan Crofts, Frances Parry‐Ford, Meera Chand, Nick Phin

**Affiliations:** ^1^ Field Service National Infection Service Public Health England Liverpool UK; ^2^ Immunisation Hepatitis, Blood Safety and Countermeasures Response National Infection Service Public Health England London UK; ^3^ Influenza Preparedness and Response Section National Infection Service Public Health England London UK; ^4^ Reference Microbiology Services National Infection Service Public Health England London UK; ^5^ Interim Deputy Director National Infection Service Public Health England London UK

**Keywords:** critical care outcomes, H1N1, influenza, pandemic, zanamivir

## Abstract

**Background:**

Aqueous zanamivir solution, an investigational product, was provided by the manufacturer on compassionate grounds for parenteral administration to severe H1N1pdm09 influenza cases during the 2009 pandemic.

**Objective:**

To describe characteristics and outcomes of UK patients receiving parenteral zanamivir therapy.

**Methods:**

Collaborators at multiple hospital sites gathered retrospective data on patients receiving aqueous zanamivir therapy between Q4 2009 and Q1 2011. We present analysis of the demographics, clinical features, treatment and outcomes of this cohort.

**Results:**

Data on 185 cases were obtained (response rate of 38%; median age 43 years; 62% male; 17% non‐Caucasian ethnic group). Most frequent co‐morbidities included cancer, immunosuppression and respiratory conditions. Most patients received intravenous zanamivir alone (90%), for durations of up to 21 days. 13% of cases had adverse effects related to zanamivir therapy. Thirty four percentage of cases died. No significant relationship was seen between mortality and timing or route of administration of aqueous zanamivir therapy.

**Conclusions:**

The response rate of this observational study of the outcomes of treatment of severe influenza was low, allowing limited conclusions to be drawn. Some potential adverse effects were noted. Clinicians should carefully consider potential risks and benefits of use of this product. New treatment options are urgently required to improve outcomes for patients with severe influenza infections.

## 1 INTRODUCTION

Most cases of pandemic H1N1pdm09 influenza experienced uncomplicated illness or asymptomatic infection.^1^ A minority of severe cases developed acute respiratory distress syndrome (ARDS) requiring respiratory support. Two neuraminidase inhibitors were licensed for influenza in the United Kingdom at the time. Oseltamivir may be administered orally and is well absorbed.^2^ Zanamivir may be administered by powder inhaler.^3^ Nebulised administration of zanamivir inhalation powder as a liquid formulation is not recommended.^4^ Few alternative antiviral formulations were available for critically ill patients with oseltamivir‐resistant infections, impaired gastric motility, malabsorption and/or gastrointestinal bleeding.

Zanamivir aqueous solution is an investigational product which may be administered via nebulisation or intravenously. At the onset of the 2009 pandemic, published evidence of the safety and effectiveness of intravenous or nebulised zanamivir aqueous solution was limited and it was not licensed for use in any country.^5^ From May 2009, zanamivir aqueous solution was made available by the manufacturer on a named‐patient basis under a global “compassionate use programme” for treatment of severe influenza where licensed antiviral drugs were not effective or practical. A total of 485 issues of aqueous zanamivir were made in the UK, to 153 sites in 113 trusts or health boards in the UK, constituting just under 40% of global use (Glaxo‐Smith‐Kline, personal communication). Although completion of case report forms was a requirement of the programme, only 29 case reports (6%) were returned to the manufacturer.

Public Health England (PHE) retrospectively collected data on use of aqueous zanamivir between October 2009 and January 2011. This report describes characteristics and outcomes of patients receiving parenteral zanamivir therapy and risk factors for severe outcome.

## 2 METHODS

All sites that had received aqueous zanamivir from the manufacturer under the compassionate use programme (CUP) between October 2009 and January 2011 were approached by PHE or via the ICS “Linkman” network of intensive care professionals. The manufacturer provided information to responding sites to identify patients who received aqueous zanamivir treatment. Cases were defined as intensive care patients for whom parenteral zanamivir aqueous solution was provided by the manufacturer under the CUP. Collaborators completed a standardised case report form providing anonymised demographic, clinical, microbiological, hospitalisation, treatment and outcome information including adverse events. Sequential Organ Failure Assessment (SOFA) scores at initiation of aqueous zanamivir therapy were provided for each case.^6^ Data collection began in March 2013 and was completed in March 2015. Follow‐up information was available for 457 of the 485 cases for whom parenteral zanamivir was issued.

The recommended adult dosage of intravenous aqueous zanamivir was 600 mg twice daily for 5 days; adjustments were required for renal impairment.^7^ Children, adolescents and pregnant women required weight‐based dosing. The recommended adult nebulised dose was 25 mg four times daily.

An adverse event was defined as any untoward medical occurrence occurring from the time of the first dose to 14 days after treatment completion in a case, irrespective of a possible causal relationship with zanamivir treatment. A serious adverse event was defined as an adverse event that results in death; is life‐threatening; requires hospitalisation or prolongation of existing hospitalisation; results in disability or incapacity; or results in a congenital anomaly or birth defect.

We summarised the characteristics of cases treated with parenteral zanamivir aqueous solution in terms of demographics; co‐morbidities, pregnancy and body weight; influenza vaccination status; clinical and radiographic findings; prior antiviral therapy; timing, route and duration of aqueous zanamivir administration; other medical treatment and respiratory support; complications and co‐infections; reported adverse events; length of stay and mortality. We calculated case fatality ratios stratified by each variable. Confidence intervals for case fatality ratios were calculated using the Wilson interval.^8^ Odds ratios for mortality with confidence intervals were calculated for categorical explanatory variables.

In all analyses, *P* values of <0.05 were considered significant. Statistical analyses were conducted using IPython^9^ and R version 3.1.3.^10^

Ethical approval of the study was granted for all NHS sites taking part in the study from the East of Scotland Research Ethics Service (EoSRES).

## 3 RESULTS

Data were returned for 185 cases (out of 457 questionnaires sent; response rate 38%). Thirty‐four sites each provided data for between 1 and 21 cases (median three cases). Data completeness was >95% for the majority of key variables.

### 3.1 Clinical/demographic characteristics

Table 1 describes the characteristics of the cases. A total of 181 cases (98%) had laboratory‐confirmed H1N1pdm09 influenza A. Of the remaining 4, 2 had influenza B and 2 had influenza A of another/unknown strain. Resistance to oseltamivir was documented for two cases (this was not part of the minimum data set). The majority of cases (81%) were reported from England. Most cases (163; 88%) were treated in calendar quarters 2009 Q4, 2010 Q4 or 2011 Q1. The age distribution of cases ranged from <1 to 74 years of age (median age 43 years). Most cases were male (62%). Female cases were significantly younger than male cases (median of 34 years, vs 48.5 for males; Wilcoxon rank sum test *P* value <0.001). Ethnic group was available for 157 cases: of these, 80% were White, 8% of African descent and 8% of Asian/Oriental descent.

**Table 1:** Characteristics of the study population and category‐specific case fatality ratios

Variable	Category	Number & percentage of patients (N = 185)		Number of deaths	Case fatality ratio	95% confidence interval for CFR	
Country	England	150	81.1	53	35.3	28.1	43.3
	Northern Ireland	10	5.4	2	20.0	5.7	51.0
	Scotland	4	2.2	1	25.0	4.6	69.9
	Wales	21	11.4	4	19.0	7.7	40.0
Age group	0‐14	17	9.2	2	11.8	3.3	34.3
	15‐24	14	7.6	4	28.6	11.7	54.6
	25‐34	27	14.7	4	14.8	5.9	32.5
	35‐44	42	22.8	16	38.1	25.0	53.2
	45‐54	42	22.8	15	35.7	23.0	50.8
	55‐64	31	16.8	15	48.4	32.0	65.2
	65+	11	6.0	4	36.4	15.2	64.6
Sex	Female	70	37.8	23	32.9	23.0	44.5
	Male	115	62.2	37	32.2	24.3	41.2
Ethnic group	African descent	13	7.0	6	46.2	23.2	70.9
	Asian/Oriental	13	7.0	6	46.2	23.2	70.9
	Caucasian	125	67.6	34	27.2	20.2	35.6
	Other	6	3.2	2	33.3	9.7	70.0
	Unknown	28	15.1	12	42.9	26.5	60.9
Any co‐morbidity		99	64.3	41	34.4	26.5	43.4
Oncology (current cancer or treatment <1 y)		44	23.8	21	47.7	33.8	62.1
Any immunocompromise (including from medication)		27	14.6	11	40.7	24.5	59.3
Any respiratory condition		25	13.5	10	40.0	23.4	59.3
Diabetes mellitus		15	8.1	6	40.0	19.8	64.3
Pregnant		15	8.1	6	40.0	19.8	64.3
Morbid obesity (BMI > 40)		10	5.4	‐	‐	‐	‐
ICU admission for respiratory failure		165	89.2	56	33.9	27.2	41.5
ICU admission for sepsis		52	28.1	17	32.7	21.5	46.2
ICU admission for cardiovascular failure		25	13.5	10	40.0	23.4	59.3
Overall SOFA score category	Low (0‐6)	39	23.1	5	12.8	5.6	26.7
	Medium (7‐8)	35	20.7	9	25.7	14.2	42.1
	High (9 + )	95	56.2	45	47.4	37.6	57.3
Chest X‐ray: Consolidation of single lobe		16	8.6	5	31.3	14.2	55.6
Chest X‐ray: Consolidation of multiple lobes		95	51.4	32	33.7	25.0	43.7
Chest X‐ray: Diffuse interstitial or reticular changes		59	31.9	22	37.3	26.1	50.0
Antiviral treatment prior to zanamivir therapy		164	88.6	51	31.1	24.5	38.5
Route of zanamivir administration	Both methods	12	6.6	3	25.0	8.9	53.2
	Intravenous	164	89.6	56	34.1	27.3	41.7
	Nebulised	7	3.8	1	14.3	2.6	51.3
Dosage adjusted due to change in renal function		27	15.5	10	37.0	21.5	55.8
Scheduled dosing interrupted during the treatment period		17	9.9	3	17.6	6.2	41.0
Zanamivir stopped permanently before the scheduled end of therapy		26	14.9	11	42.3	25.5	61.1
Renal impairment		33	20.0	15	45.5	29.8	62.0
Cardiovascular impairment		28	17.1	16	57.1	39.1	73.5
Hepatic impairment		22	13.6	9	40.9	23.3	61.3
Haematological impairment		18	11.1	7	38.9	20.3	61.4
Neurological impairment		14	8.5	6	42.9	21.4	67.4
Gastrointestinal impairment		9	5.5	3	33.3	12.1	64.6
Serious adverse event related to zanamivir (SAE)		13	7.0	5	38.5	17.7	64.5
Non‐serious adverse event related to zanamivir (AE)		14	7.6	1	7.1	1.3	40.2
Systemic antibacterial therapy		178	96.2	57	32.0	25.6	39.2
Inotrope support		126	68.1	48	38.1	30.1	46.8
Continuous renal replacement therapy/haemodialysis		72	38.9	34	47.2	36.1	58.6
Corticosteroid therapy		94	50.8	41	43.6	34.0	53.7
Endotracheal mechanical ventilation		164	88.6	56	34.1	27.3	41.7
ECMO		26	14.1	10	38.5	22.4	57.5
CPAP		55	29.7	19	34.5	23.4	47.7
BiPAP		27	14.6	10	37.0	21.5	55.8
ARDS		80	43.2	36	45.0	34.6	55.9
Bacterial pneumonia		75	40.5	26	34.7	24.9	45.9
Sepsis		73	39.5	27	37.0	26.8	48.5
Complications of pneumonia		35	18.9	17	48.6	33.0	64.4

The recorded date of hospital admission preceded the recorded date of onset of influenza symptoms in 28 cases (15%): by more than 7 days in most cases (64%) and by two or fewer days in only five cases (18%). Excluding cases with onset 2 days or less after admission, up to 12% (23/185) of infections, may have been acquired nosocomially.

Frequent co‐morbidities included current cancer or cancer treatment in the previous year (24%; most were diagnosed with leukaemia or lymphoma); immunosuppression (15%); respiratory conditions (14%, including asthma, chronic obstructive pulmonary disease or unspecified chronic lung disorders); and diabetes mellitus (8%). Ten patients were recorded as morbidly obese.

Fifteen cases (8%) were pregnant; of these, 12 (80%) were in the third trimester.

Information on influenza vaccination status was available for only five cases (2 had received vaccination against H1N1pdm09; for 3, the vaccine was not specified).

The median SOFA score at the time of initiation of zanamivir therapy was 9 (lower and upper quartiles 7 and 13). Higher scores were recorded for the respiratory, neurological and cardiovascular domains of the SOFA score (Friedman rank sum test *P* value <0.0001).

Common chest X‐ray abnormalities were as follows: consolidation of multiple lobes (49%), diffuse interstitial or reticular changes (32%) and consolidation of a single lobe (9%). Eight cases (4%) had a normal chest X‐ray.

A total of 92 cases (52%) developed microbiological evidence of secondary infection. Microbiology results were available for 85 cases; the most common pathogens were Enterobacteriaceae or unspecified Gram‐negative organisms (27%), *Pseudomonas* sp. (21%), streptococci (19%), *Candida*/yeast (12%) and *Staphylococcus aureus* (12%).

### 3.2 Treatment

Most cases (164 cases; 89%) received antiviral therapy prior to aqueous zanamivir therapy (where specified, oseltamivir was given for all but two cases, who received inhaled zanamivir therapy). Duration of prior antiviral therapy was reported for 73%: 72 cases (60%) received therapy for 5 days or less, 40 cases (33%) for 6‐14 days and eight cases (7%) for greater than 14 days.

The most common reasons for admission to intensive care were respiratory failure (89%), sepsis (28%) and cardiovascular failure (14%).

Most cases (90%) received intravenous zanamivir therapy alone, the remainder receiving nebulised zanamivir therapy alone or zanamivir via both routes. The duration of intravenous zanamivir therapy ranged from 1 to 21 days (median 6 days). The duration of nebulised zanamivir therapy ranged from 1 to 10 days (median 5 days). Dosage was adjusted for renal function for 27 cases (16%).

Median overall SOFA scores were higher for cases given intravenous aqueous zanamivir therapy (10, vs 4 and 5 for cases treated with nebulised therapy or via both routes respectively; *P* = 0.0005 by Kruskal‐Wallis test).

In addition to zanamivir therapy, cases received systemic antibacterial therapy (96%), inotrope support (68%), continuous renal replacement therapy/haemodialysis (39%) and/or corticosteroid therapy (51%). A total of 164 cases (89%) received endotracheal mechanical ventilation (EMV), for a median duration of 16 days; of these, 26 cases (16% of those receiving EMV) received extra‐corporeal mechanical oxygenation (ECMO), for a median duration of 18 days. Of cases who did not receive EMV or ECMO, 8 received non‐invasive continuous positive airway pressure (CPAP) ventilation and 2 received bi‐level positive airway pressure (BiPAP) ventilation.

### 3.3 Complications and adverse events

Common complications of influenza infection included acute respiratory distress syndrome (43%), bacterial pneumonia (41%) and sepsis (40%). One or more manifestations of renal, cardiovascular, hepatic, haematological, neurological and gastrointestinal compromise were seen in 70 cases (38%) during zanamivir therapy.

Scheduled dosing was interrupted during the treatment period for 17 cases (10%) and was stopped before the scheduled end of therapy in 26 cases (15%). The reason for early discontinuation of treatment was recorded for 24 cases; the reason was physician discretion for 15 cases (63%) and suspected adverse events for nine cases (38%).

A total of 163 adverse events were recorded for 81 cases (44% of all cases), of which 86 were described as serious. Twenty‐four cases (13%) had adverse events which were reported as temporally related to zanamivir therapy (of which 18 events were recorded as serious, including three deaths, five events of hepatic dysfunction (including one event of hepatic failure) and four acute renal failure events).

### 3.4 Mortality

Outcomes were recorded for 175 cases (95%). Of these, 97 cases (55%) recovered, 18 cases (10%) recovered with permanent sequelae and 60 cases (34%) died. Death certificate data were available for all 60 deaths. Influenza was recorded as a primary cause of death for 37 cases (62%) and pneumonia, pneumonitis, bronchopneumonia or acute respiratory distress syndrome of unspecified cause for a further nine cases (15%). For one patient, influenza was a secondary cause of death. Influenza or compatible syndromes were not recorded among the causes of death for five cases (8%).

Age group (highest relative mortality in the 55‐64 years age group), cardiovascular impairment, complications of pneumonia, renal replacement therapy, corticosteroid therapy and high SOFA scores were significantly associated with mortality (Table 2). Non‐serious adverse events temporally related to zanamivir were associated with significantly reduced mortality, and serious adverse events temporally related to zanamivir were not significantly associated with mortality.

**Table 2:** Single variable analysis of possible risk factors for mortality

Variable (base: No, unless specified)	Category	Proportion of fatal cases with this exposure (%)	Proportion of survivors with this exposure (%)	Odds ratio	95% confidence interval for OR		*P* value
ARDS		60.0	35.2	2.8	1.4	5.5	<0.01
Age group (base: <15 y)	15‐24 y	66.7	40.0	3.0	0.3	37.8	0.37
	25‐34 y	66.7	60.5	1.3	0.2	16.0	1.00
	35‐44 y	88.9	63.4	4.6	0.9	45.9	0.09
	45‐54 y	88.2	64.3	4.2	0.8	41.6	0.11
	55‐64 y	88.2	51.6	7.0	1.2	71.1	0.03
	65+ y	66.7	31.8	4.3	0.5	55.0	0.17
Antiviral treatment prior to zanamivir therapy		87.9	91.1	0.7	0.2	2.3	0.68
Any respiratory condition		16.7	12.0	1.5	0.5	3.8	0.52
Any immunocompromise (including by medication)		18.3	12.8	1.5	0.6	3.8	0.44
Bacterial pneumonia		43.3	39.2	1.2	0.6	2.3	0.71
Cardiovascular failure (reason for ICU admission)		16.7	12.0	1.5	0.5	3.8	0.52
Cardiovascular impairment		30.8	10.7	3.7	1.5	9.4	<0.01
Complications of pneumonia		28.3	14.4	2.4	1.0	5.3	0.04
Chest X‐ray: Consolidation of single lobe		8.3	8.8	0.9	0.2	3.1	1.00
Chest X‐ray: Consolidation of multiple lobes		53.3	50.4	1.1	0.6	2.2	0.83
Continuous renal replacement therapy/haemodialysis		56.7	30.4	3.0	1.5	6.0	<0.01
Corticosteroid therapy		68.3	42.4	2.9	1.5	6.0	<0.01
Diabetes mellitus		10.0	7.2	1.4	0.4	4.8	0.57
Chest X‐ray: Diffuse interstitial or reticular changes		36.7	29.6	1.4	0.7	2.8	0.43
Dosage adjusted due to change in renal function		17.9	14.4	1.3	0.5	3.3	0.72
ECMO		16.7	12.8	1.4	0.5	3.5	0.63
Endotracheal mechanical ventilation		93.3	86.4	2.2	0.7	9.4	0.25
Ethnic group (base: Caucasian)	African descent	15.0	7.1	2.3	0.6	8.6	0.20
	Asian/Oriental	15.0	7.1	2.3	0.6	8.6	0.20
	Other	5.6	4.2	1.3	0.1	9.8	0.67
	Unknown	26.1	15.0	2.0	0.8	5.0	0.16
Gastrointestinal impairment		6.0	5.3	1.1	0.2	5.6	1.00
Haematological impairment		14.3	9.7	1.5	0.5	4.7	0.57
Hepatic impairment		17.3	11.8	1.6	0.5	4.3	0.48
Inotrope support		80.0	62.4	2.4	1.1	5.5	0.03
Morbid obesity (BMI > 40)		0.0	8.0	0.0	0.0	0.9	0.03
Neurological impairment		11.8	7.1	1.8	0.5	6.1	0.37
BiPAP		16.7	13.6	1.3	0.5	3.2	0.74
CPAP		31.7	28.8	1.1	0.6	2.3	0.82
Non‐serious adverse event related to zanamivir (AE)		1.7	10.4	0.15	<0.1	1.0	0.04
Oncology (current cancer or treatment <1 y)		35.0	18.4	2.4	1.1	5.1	0.02
Overall SOFA score category (base: high/9 + )	Low (0‐6)	10.0	40.5	0.2	0.0	0.5	<0.01
	Medium (7‐8)	16.7	34.2	0.4	0.1	1.0	0.04
Route of zanamivir administration (base: nebulised)	Both methods	75.0	60.0	2.0	0.1	122.1	1.00
	Intravenous	98.2	94.7	3.1	0.4	145.5	0.43
Pregnant		27.3	24.3	1.2	0.3	4.5	1.00
Renal impairment		28.8	15.9	2.1	0.9	5.0	0.09
Respiratory failure		93.3	87.2	2.1	0.6	8.8	0.32
Scheduled dosing interrupted during the treatment period		5.5	12.0	0.4	0.1	1.6	0.29
Sepsis (reason for ICU admission)		45.0	36.8	1.4	0.7	2.7	0.36
Sepsis (complication)		28.3	28.0	1.0	0.5	2.1	1.00
Serious adverse event related to zanamivir (SAE)		8.3	6.4	1.3	0.3	4.9	0.76
Sex (base: Female)	Male	61.7	62.4	1.0	0.5	1.9	1.00
Systemic antibacterial therapy		95.0	96.8	0.6	0.1	4.4	0.68
Zanamivir stopped permanently before the scheduled end of therapy		19.3	12.8	1.6	0.6	4.1	0.37

Among those who died median time to zanamivir treatment was 13 days, compared with 10 days for those who survived (*P* = 0.22).

## 4 DISCUSSION

This is the largest observational study of a cohort of patients with severe influenza treated with parenteral zanamivir therapy reported to date. The co‐morbidities and other risk factors for severe influenza described in this cohort reflect the overall epidemiology of the H1N1pdm09 pandemic influenza virus, which disproportionately impacted younger adult age groups and those with risk factors including immunosuppression, morbid obesity and pregnancy.^1^

However, the response rate was low and it is not possible to draw conclusions about the effect of parenteral zanamivir from this study. Some adverse effects were noted. Cases received aqueous zanamivir therapy because of disease progression following treatment with oral or nasogastric oseltamivir rather than because of oseltamivir‐resistant disease. Aqueous zanamivir treatment was commonly provided late in the course of the disease, after admission to intensive care for management of respiratory and/or multi‐organ failure and treatment of co‐infections. Most cases received intravenous zanamivir therapy, while a minority of less severe cases tended to receive nebulised zanamivir therapy or a combination. Mortality was high and no significant relationship of mortality to timing or route of administration of aqueous zanamivir therapy was detected. A minority of cases experienced adverse events, some serious, in relation to parenteral zanamivir therapy, but as there was no control group, it was not possible to distinguish the effects of zanamivir from progression of disease, complications or adverse effects of other concomitant treatment.

There is limited previous published evidence of the use of parenteral aqueous zanamivir. A number of individual case reports describing the use of intravenous zanamivir in the treatment of severe influenza cases have been published.^11^, ^12^, ^13^, ^14^, ^15^ Fraaij et al^16^ reported a retrospective study of the use of intravenous zanamivir in 26 severe influenza patients in the Netherlands, noting possible reductions in viral load in patients treated earlier, which did not reduce mortality, and concluding that late use of intravenous zanamivir may be of limited effectiveness. Chan‐Tack et al^17^ summarised the use of intravenous zanamivir as part of the United States Food and Drugs Administration's Emergency Investigational New Drugs application process and concluded that randomised clinical trials were required to identify the benefits and risks of intravenous zanamivir for severe influenza cases. In both studies, a substantial proportion of cases were noted to have prior immunosuppression, and only a minor proportion were known to have oseltamivir‐resistant infections, as in our study. Most cases had received prior treatment with oseltamivir, a drug which is well absorbed and has the same mode of action as zanamivir. Zanamivir has previously been reported as well tolerated, but most published previous evaluation has been of the inhaled powder formulation, which leads to low levels of systemic absorption, or in healthy volunteers.^3^, ^18^ Intravenous administration of aqueous zanamivir results in higher systemic absorption^19^ which could result in more frequent or severe adverse events. It is of note that courses of aqueous zanamivir treatment were commonly prolonged beyond the recommended 5 days. Further evaluation of the tolerability and safety of aqueous zanamivir for severe influenza requires further clinical trials and post‐marketing surveillance. Clinicians need to make careful assessment of the potential risks and benefits of use of this product.

Pebody et al^20^ reported an analysis of mortality among cases of influenza A/H1N1pdm09 in the UK from April 2009 to March 2010, which has a degree of overlap with the period of this study. This analysis reported an overall estimated symptomatic case fatality ratio of 0.4 per 1000 clinical cases, finding a similar age distribution among severe or fatal cases to this study. The relative risk of mortality was higher in cases in clinical risk groups, particularly those with underlying immunosuppression. Our analysis adds to this by showing that in a subset of severe UK cases during this period, with a high prevalence of risk factors for severe disease, the main risk factor for mortality was a current diagnosis of cancer or recent cancer treatment, after adjustment for demographics and markers of severity of disease.

After adjustment for age and other predictors of mortality, female sex was associated with greater mortality in this study, even excluding pregnant women from analysis. Crude overall mortality was similar between sexes, but female cases were significantly younger than male cases; greater relative mortality in females was apparent after adjustment for age and other predictors of mortality. There are limited published data on sex differences in mortality among severe influenza H1N1pdm09 cases from comparable populations. Oliveira et al^21^ reported similar case fatality ratios for males and females in a large Brazilian cohort, but did not standardise for age. Archer et al^22^ reported a female predominance (59%) among South African fatalities during the early pandemic, in a population with significant co‐morbidities including HIV infection. Possible interpretations of the greater mortality among females in our study include: greater virulence of H1N1pdm09 for women at risk of severe influenza disease; sex differences in response to antivirals or other treatment; bias due to under‐recording of pregnancy or other key risk factors; and/or confounding by other factors not captured as part of this study.

Up to 12% of cases in this study may have arisen from nosocomial acquisition of influenza infection, often several days or more after admission. Our estimate is higher than the 2% acquired nosocomially among 1520 patients admitted to hospitals in the United Kingdom with H1N1pdm09 pandemic influenza, as reported by Enstone et al^23^ This finding of greater nosocomial acquisition among severe influenza cases than among the population of all hospitalised influenza cases could be explained by a higher prevalence of co‐morbidities among severe influenza cases, leading to lengthier prior hospital exposure and/or greater susceptibility to infection. It underlines the importance of careful infection control and other measures, such as staff vaccination, to reduce exposure of patients to influenza.

This study has a number of important limitations. Recruitment was voluntary and the response rate was low. Data collection was retrospective, albeit based on contemporaneous patient records. The indication for aqueous zanamivir treatment was a decision by the treating clinicians and was not standardised. No comparison data were available from a control group or other sources. No information on intermediate outcomes such as sequential viral load was available.

In the event of a future potential pandemic, information on novel treatments needs to be collected and analysed in real time in order to inform the response to a pandemic, using randomised, controlled trials where possible. Future evaluations of investigational products during influenza pandemics will require standardised collection of high‐quality data to inform the evidence base, ideally planned in advance allowing for adequate staff training, and undertaken prospectively; a challenge that is currently being addressed by the PREPARE^24^ and ISARIC^25^ projects. New treatment options are urgently required to improve outcomes for patients with severe and life‐threatening influenza infections.
